# Molecular Mechanisms of Autophagy Regulation in Plants and Their Applications in Agriculture

**DOI:** 10.3389/fpls.2020.618944

**Published:** 2021-02-16

**Authors:** Jia-Jian Cao, Chen-Xu Liu, Shu-Jun Shao, Jie Zhou

**Affiliations:** ^1^Zhejiang Provincial Key Laboratory of Horticultural Plant Integrative Biology, Department of Horticulture, Zhejiang University, Hangzhou, China; ^2^Key Laboratory of Horticultural Plants Growth, Development and Quality Improvement, Agricultural Ministry of China, Hangzhou, China

**Keywords:** agricultural applications, autophagy, autophagy-related genes, epigenetic regulation, phytohormone, transcription factor, ubiquitin–proteasome system

## Abstract

Autophagy is a highly conserved cellular process for the degradation and recycling of unnecessary cytoplasmic components in eukaryotes. Various studies have shown that autophagy plays a crucial role in plant growth, productivity, and survival. The extensive functions of plant autophagy have been revealed in numerous frontier studies, particularly those regarding growth adjustment, stress tolerance, the identification of related genes, and the involvement of metabolic pathways. However, elucidation of the molecular regulation of plant autophagy, particularly the upstream signaling elements, is still lagging. In this review, we summarize recent progress in research on the molecular mechanisms of autophagy regulation, including the roles of protein kinases, phytohormones, second messengers, and transcriptional and epigenetic control, as well as the relationship between autophagy and the 26S proteasome in model plants and crop species. We also discuss future research directions for the potential application of autophagy in agriculture.

## Plain Language Summary

In the last two decades, numerous studies have reported that the autophagy pathway is precisely regulated in plants. We summarize recent progress on the molecular mechanisms of autophagy regulation in plants and discuss future research directions for the potential application of autophagy in agriculture.

## Introduction

Autophagy, literally meaning “self-eating,” is a highly conserved cellular process for the degradation and recycling of unnecessary cytoplasmic components, including unnecessary proteins, damaged nuclear fragments, dysfunctional complexes, and even whole organelles, in eukaryotes. Three distinct but not mutually exclusive types of autophagy have been reported in plants, including macroautophagy, microautophagy, and mega-autophagy (Marshall and Vierstra, 2018). Macroautophagy is characterized by the sequestration of cellular cargos by double-membrane structures called autophagosomes, which fuse with the vacuole for digestion and recycling. Macroautophagy is the best-characterized form of autophagy; therefore, it is simply regarded as autophagy. Macroautophagy can be either nonselective or selective. Nonselective macroautophagy, stimulated by nutritional deficiency, normally refers to random bulk protein degradation, whereas selective macroautophagy specifically removes specific components and involves the recognition of autophagy substrates by dedicated receptors (Johansen and Lamark, 2020). Based on the specific recognition and degradation of organelles or pathogens, the forms of selective autophagy are named mitophagy for mitochondria degradation, chlorophagy for chloroplast degradation, reticulophagy for endoplasmic reticulum (ER) degradation, and xenophagy for intracellular pathogen degradation (Abdrakhmanov et al., 2020). In contrast, microautophagy is the direct uptake of cytoplasmic materials into the vacuole by invagination or protrusion of the tonoplast, such as anthocyanin aggregate transport from the cytosol to the vacuole, and these materials are directly engulfed by the vacuolar membrane, eventually becoming free in the vacuolar lumen (Chanoca et al., 2015). Mega-autophagy is an extreme autophagic process accomplished by permeabilization or rupture of the vacuolar membrane (van Doorn and Woltering, 2005). Mega-autophagy appears to be the most common type during programmed cell death (PCD), which occurs during development or in response to pathogenic invasion as in the case of xylem formation in Arabidopsis (Kwon et al., 2010), senescence (Liu and Bassham, 2012), and plant–pathogen interactions (Leary et al., 2019). Additionally, chaperone-mediated autophagy (CMA), which is a selective form of autophagy, occurs in most mammalian cells through cytosolic chaperone proteins that target substrates, but no functional counterparts have been identified in plants.

The genetic machinery of macroautophagy (hereafter termed as autophagy) has been systematically deciphered by the identification and functional analysis of over 40 autophagy-related (*ATG*) genes in eukaryotes (Furukawa et al., 2019). Plant genomes encode multiple orthologs of identified ATG members in yeast and mammals. These ATG proteins are traditionally divided into four protein complexes, including the ATG1 complex with scaffold protein ATG11/17 for the initiation of autophagy, transmembrane core protein ATG9 with ATG2/ATG18 for nucleation and phagophore expansion, the phosphatidylinositol 3-kinase (PI3K) complex for phagophore decoration, and ATG8/12 conjugation systems for autophagosome maturation (Tang and Bassham, 2018; Zhuang et al., 2018). Many excellent reviews have discussed the functions and categories of *ATG* genes (Bassham et al., 2006; Vanhee and Batoko, 2011; Michaeli et al., 2016; Antonioli et al., 2017; Galluzzi et al., 2017); thus, these topics are not covered here in detail.

**FIGURE 1:**
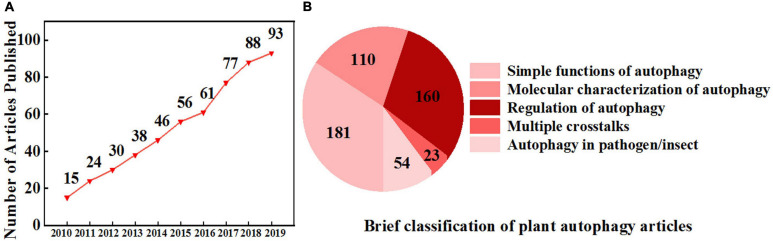
Increasing interest in plant autophagy. **(A)** The number of research articles published on autophagy in plants from 2010 to 2019. Information was retrieved from the Web of Science with the topic “plant autophagy” refined by the database “Web of Science Core Collection.” **(B)** A brief classification of plant autophagy articles from 2010 to 2019 based on **(A)** by manual division.

In the first two decades of the current century, research on autophagy in plants expanded rapidly and explored the elements and the molecular mechanisms of autophagy, multiple ultrastructures involved in autophagy, and significant roles of autophagy in plant development and environmental responses (Figure 1). Compared to previous extensive functional research on autophagy, research on the regulatory mechanisms of the autophagy pathway is still lagging. Hence, a comprehensive review outlining the recent research on autophagy regulators in plants is needed. In the present review, we summarize recent advances in research on the molecular regulation of autophagy in plants, including the roles of protein kinases, phytohormones, second messengers, and transcriptional and epigenetic regulators. We also discuss the connection and distinction between autophagy and the 26S proteasome and the applications and prospects of autophagy in agriculture.

## Regulators of Autophagy Induction

**FIGURE 2:**
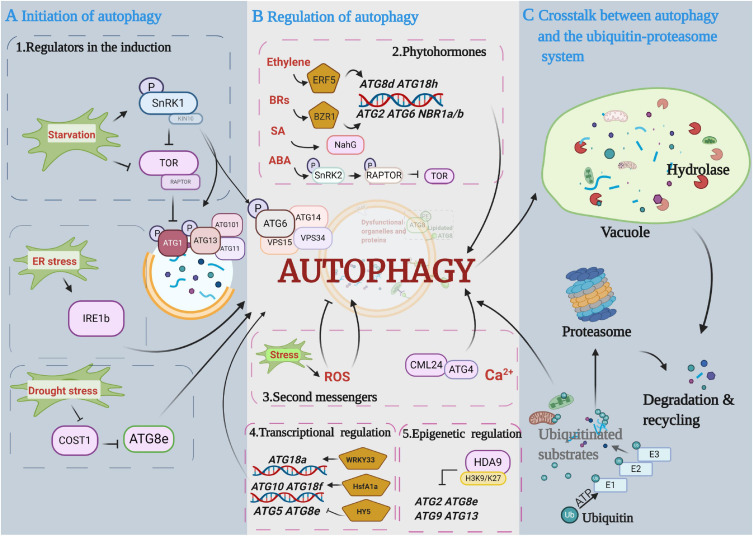
Diagram depicting the main proposed events in the autophagy pathway. **(A)** Initiation of autophagy. Autophagy is initiated by protein kinases and developmental and environmental signals. Autophagy induction and nucleation involve the autophagy-related gene 1 (ATG1) complex that is negatively controlled by target of rapamycin (TOR) kinase and activated by sucrose nonfermenting-1-related protein kinase 1 (SnRK1) kinase. **(B)** Regulation of autophagy in autophagosome expansion and maturation. Multiple regulatory mechanisms are involved in autophagy processes, including phytohormones, secondary messengers, and transcriptional and epigenetic regulators. **(C)** Crosstalk between autophagy and the ubiquitin-proteasome pathway (UPS). Ubiquitinated substrates are precisely identified and degraded by the UPS and autophagy pathways, respectively. This figure was created by BioRender (https://biorender.com/).

The conserved hierarchical steps of autophagosome formation include the following two major initiation complexes in plants (Figure 2A): (1) the ATG1 kinase complex, including ATG1, ATG13, ATG11, and ATG101, which responds to nutritional signals, and (2) the PI3K complex, which is characterized by two heterotetramers, namely, complex I and complex II, and remodels autophagic membranes (Zhuang et al., 2018). Complex I includes vacuolar protein sorting 34 (VPS34) and the accessory proteins VPS15, ATG6, and ATG14, whereas in complex II, ATG14 is replaced with VPS38 (Liu et al., 2020). ATG1 is the main switch activating autophagy, and it is normally regulated by upstream kinases (Hurley and Young, 2017). Target of rapamycin (TOR) and sucrose nonfermenting-1-related protein kinase 1 (SnRK1) are two evolutionarily conserved protein kinase complexes that play central and antagonistic roles in the initiation of autophagy (Rodriguez et al., 2019). TOR inhibits and SnRK1 activates ATG1 kinase under nutrient starvation and stress conditions, respectively. Mammals and yeast have TOR complex 1 (TORC1) and TORC2; however, only TORC1 has been identified in plants. Plant TORC1 includes the central element TOR kinase, two regulatory-associated protein of TOR (RAPTOR) subunits and lethal with sec thirteen 8 (LST8). TOR-dependent control of autophagy was first studied in animals. It has been reported that TOR promotes hyperphosphorylation of ATG13 to decrease its affinity to ATG1 and represses unc-51-like autophagy activating kinase 1 (ULK1, ATG1 homolog) activity through direct dephosphorylation of Ser757 under nutrient sufficiency (Kamada et al., 2010; Kim et al., 2011). Similar to the regulation in animals, the autophagy-initiating ATG1/ATG13 kinase complex is negatively regulated by the TOR complex in plants (Suttangkakul et al., 2011). During nutrition deprivation, inhibition of TOR signaling leads to dephosphorylation of ATG13 and hyperphosphorylation of ATG1a to activate the autophagy pathway (Li and Vierstra, 2012). Moreover, the mammalian homolog of SnRK1, AMP-activated protein kinase (AMPK), was previously reported to promote autophagy by directly activating ULK1 through phosphorylation of Ser317 and Ser777 (Kim et al., 2011). It has been recently reported that plant SnRK1 activates autophagy *via* inhibition of the TOR signaling pathway or direct activation of ATG proteins. For instance, Arabidopsis SnRK1 subunit KIN10 has been shown to interact with RAPTOR in the cytosol and to phosphorylate RAPTOR by kinase assays, suggesting that SnRK1 phosphorylation of RAPTOR represses TOR complex activity to activate autophagy in plants (Nukarinen et al., 2016; Pu et al., 2017b). KIN10 has also been shown to directly phosphorylate ATG1, and overexpression of Arabidopsis *KIN10* enhanced the phosphorylation of ATG1 under carbon starvation and activated the autophagy signaling pathway (Chen L. et al., 2017). Furthermore, in addition to regulating the ATG1 complex, SnRK1 can also directly phosphorylate ATG6 to activate the PI3K complex by sensing nutritional status (Huang X. et al., 2019). Thus, SnRK1-mediated activation of the PI3K complex is a possible alternative route of autophagy initiation when the ATG1 initial complex is under prolonged fixed-carbon starvation in plants.

In addition to nutrient starvation-induced autophagy, multiple types of stress-induced autophagy are initiated through TOR/SnRK1-independent signaling pathways in plants. For instance, one potential regulator is inositol-requiring enzyme 1b (IRE1b), a dual protein kinase and ribonuclease, which indirectly activates autophagy by degrading the RNA transcripts of factors that interfere with the induction of autophagy under ER stress (Bao et al., 2018). Constitutively stressed 1 (COST1) has been reported as a possible negative regulator of autophagy through direct interaction with ATG8e in plants. Arabidopsis *cost1* mutants exhibited strong drought tolerance with constitutive induction of broad expression of typical stress-responsive genes and autophagy initiation (Bao and Bassham, 2020). Furthermore, the stress-responsive protein SnRK2, a core abscisic acid (ABA) signaling kinase, appears to inhibit TOR activity and thus indirectly induces autophagy (Wang et al., 2018). However, whether SnRK2 can directly interact with ATG proteins remains to be further explored.

The regulation of selective autophagy initiation is seldom studied in plants. In mammals, it has been previously demonstrated that autophagic processes could be mediated independently by cargo receptors, such as nuclear dot protein 52 and TANK-binding kinase 1 (NDP52/TBK1), which initiate autophagy by recruiting ULK1 to cargo in the absence of microtubule-associated protein 1 light chain 3 (LC3, the mammalian homolog of ATG8) in HeLa cells (Vargas et al., 2019). A major cargo receptor p62/Sequestosome-1 (SQSTM1) binding to the scaffolding protein FIP200 (homolog of yeast ATG17) can promote autophagosome formation *via* the interaction between disordered residues 326–380 in p62 and the C-terminal region of FIP200 in HAP1 cells (Turco et al., 2019).

## Phytohormones

Phytohormones are critical endogenous molecules that regulate physiological and molecular reactions during plant growth and development and during the stress response. A growing body of evidence suggests that plant autophagy is regulated by phytohormones (Figure 2B). The application of exogenous hormones can directly regulate autophagy initiation. For instance, benzothiadiazole (BTH), a functional analog of salicylic acid (SA), can induce autophagy through the SA signal transducer Nonexpresser of PR genes 1 (NPR1). BTH-induced rapid accumulation of autophagosomes was compromised in *npr1* mutants (Yoshimoto, 2010; Munch et al., 2014). Overexpression of the *NahG* gene, which encodes a bacterial SA hydroxylase that converts SA to an inactive form, clearly suppressed SA-mediated early senescence in *atg* mutants (Yoshimoto, 2010). Furthermore, zeatin, a natural cytokinin (CTK), inhibited Arabidopsis primary root growth and reduced autophagy in root epidermis cells (Slavikova et al., 2008). Phytohormone response factors also participate in the regulation of autophagy. Ethylene response factor 5 (ERF5) directly binds to the *ATG8d* and *ATG18h* gene promoters and induces the transcription of both genes and autophagy in tomato under drought stress (Zhu et al., 2018). Meanwhile, the tomato brassinosteroid (BR) signaling transcription factor (TF) brassinazole resistance 1 (BZR1) also directly binds to the *ATG2* and *ATG6* gene promoters, and *BZR1*-overexpressing plants showed enhanced tolerance to nitrogen starvation along with an increase in *ATG* gene expression and autophagy (Wang et al., 2019). In addition to direct regulation, there are some indirect connections between autophagy and phytohormones. An increase in intercellular ABA can reduce the persulfidation of ATG4 by hydrogen sulfide and subsequently increase ATG4 protease activity and the formation of autophagosomes (Laureano-Marin et al., 2020). Other connections exist between autophagy and ABA as mentioned above; for example, ABA activates SnRK2 kinases, which phosphorylates RAPTOR and represses TOR activity to induce autophagy under osmotic stress (Wang et al., 2018). In addition, it has been reported that SnRK2 kinases have dual roles in the regulation of SnRK1 during plant growth and stress (Belda-Palazon et al., 2020; Laureano-Marin et al., 2020). Under optimal conditions, SnRK2s, together with the harbored type 2C phosphatases (PP2Cs), form “repressor complexes” that sequester SnRK1 to promote plant growth. Under stress conditions, stress-induced ABA helps disassemble SnRK2s and PP2C-containing SnRK1 repressor complexes, and thus, the released SnRK2s and SnRK1α trigger stress responses (Belda-Palazon et al., 2020). These results led us to hypothesize that SnRK2 not only directly represses TOR activity but also regulates SnRK1 activation to induce autophagy under stress conditions. In addition, mounting evidence also suggests that phytohormone biosynthesis and signal transduction pathways are affected by autophagy. The expression of phytohormone biosynthetic genes and the levels of endogenous phytohormones are changed in *atg* mutants. Higher auxin levels were observed in Arabidopsis *atg5* and *atg7* root tips, with enhanced root meristem activities on 3% glucose-based media. Moreover, significantly lower auxin levels were observed in *atg* mutants on media lacking glucose than in wild-type seedlings, indicating that auxin biosynthesis is changed in *atg* mutants under different growth conditions (Huang L. et al., 2019). Endogenous levels of active forms of gibberellins (GAs, including GA_1_, GA_4_, and GA_7_) and CTK (*trans-*zeatin) were significantly lower in the anthers of rice *atg7* mutants, which show limited dehiscence and a sterility phenotype (Kurusu et al., 2017). Thus, the loss of autophagy function results in phytohormone and cellular metabolism disorders. Furthermore, phytohormone signal transduction is also connected to autophagy. The BR core signaling element BZR1 can be degraded through an autophagy-dependent pathway under sugar starvation. Treatment with the autophagy inhibitor 3-methyladenine (3MA) prevents estradiol-induced BZR1 degradation (Zhang et al., 2016). The Arabidopsis BR master regulator BRI1-EMS suppressor 1 (BES1) interacts with the ubiquitin receptor protein dominate suppressor of KAR2 (DSK2) and is targeted by autophagy for degradation during stress *via* the interaction of DSK2 with ATG8 (Nolan et al., 2017). EXO70D-mediated selective autophagy can target the negative regulators of CTK signaling, type-A response regulators (type-A ARR), for degradation in Arabidopsis roots (Acheampong et al., 2020). All of these results demonstrate that selective receptor-mediated autophagy could precisely modulate phytohormone signaling.

Moreover, several hormone-related proteins, including indole-3-acetic acid inducible 17 (IAA17), IAA27, polar auxin transport inhibitor sensitive 1 (PIS1), and ABI5-binding protein 3 (AFP3), contain an ATG8-interacting motif (AIM) or ubiquitin-interacting motif (UIM) and interacted with ATG8 in a yeast two-hybrid assay *in vitro*; thus, they are potential substrates for autophagy (Marshall et al., 2019). Therefore, autophagy may precisely regulate multiple phytohormone signaling pathways by degrading the signal components.

## Second Messengers

Autophagy, as a process of cytoplasmic component recycling and reuse, is also regulated by second messengers, such as reactive oxygen species (ROS), nitric oxide (NO), Ca^2+^, and the cyclic nucleotides cAMP and cGMP (Figure 2B).

Under abiotic and biotic stresses, ROS, including superoxide anions (O_2_^–^), hydrogen peroxide (H_2_O_2_), singlet oxygen (^1^O_2_), and hydroxyl radical (OH), have been thought to play a dual role in plant biology (Mittler, 2017), as they can operate as important second messengers that trigger several signaling cascades at low levels and cause severe oxidative damage to DNA, RNA, proteins, and cellular membranes at high levels (Medeiros et al., 2020). ROS can modulate autophagy by targeting upstream factors or key autophagy genes. Redox signals directly modulate the kinase activity of the autophagy upstream regulator SnRK1; for example, it has been demonstrated that Arabidopsis KIN10 activity is strongly dependent on the redox status *in vitro* and that this redox sensitivity is conferred by a single cysteine residue (Wurzinger et al., 2017). Furthermore, ATG4 proteases were inhibited by ROS to ensure lipidation of ATG8 and autophagy progression in Arabidopsis and *Chlamydomonas reinhardtii* under stress conditions (Woo et al., 2014; Perez-Perez et al., 2016). NO signaling is related to autophagy through its master regulator S-nitrosoglutathione reductase 1 (GSNOR1). The conformation of GSNOR1 can be changed to expose its AIM by *S*-nitrosylation at the Cys10 residue, after which it is bound by ATG8 and degraded in an AIM-dependent manner during hypoxia responses in Arabidopsis (Zhan et al., 2018). Moreover, highly reactive and toxic oxidative species cause oxidation and denaturation of cellular proteins, which are specific substrates for autophagic degradation. For instance, more oxidative proteins were aggregated in autophagy-impaired plants under oxidative stress (Xiong et al., 2007). Exogenous H_2_O_2_ application-damaged peroxisomes were selectively degraded by autophagy in Arabidopsis (Shibata et al., 2013). Unnecessary or damaged peroxisomes can be degraded by selective autophagy, called pexophagy, which is a crucial quality control system of peroxisomes in plant cells (Borek et al., 2019; Su et al., 2020). However, how peroxisomes are marked for degradation in plants is not yet clear (Su et al., 2020). Moreover, aggregation of peroxisomes and high levels of ROS accumulation are observed in Arabidopsis *atg* mutants, which leads to disorders of guard cell ROS homeostasis and stomatal defects (Yamauchi et al., 2019). In addition, autophagy was decreased in mitochondrial alternative oxidase 1a (AOX1a) RNAi tomato plants with increased levels of H_2_O_2_ (Zhu et al., 2018), and increased catalase aggregation occurred in Arabidopsis selective autophagy cargo receptor *next to BRCA1 gene 1* (*nbr1*) mutants under heat stress (Zhou et al., 2014b).

Unlike the large amounts of ROS and autophagy research, reports on the connection between calcium signaling and autophagy in plants are limited, though this connection has been extensively reported in animals. Previous results from an animal study showed that intracellularly sequestered calcium could induce autophagy in hepatocytes (Gordon et al., 1993). Subsequent studies showed that Ca^2+^ signaling is an essential component of the AMPK-dependent autophagy pathway. AMPK could be activated by Ca^2+^/calmodulin-dependent protein kinase kinase-β (CaMKKβ) in insulinoma cells, providing a further association between Ca^2+^ signaling and autophagy (Witters et al., 2006). In Arabidopsis, calmodulin-related protein 24 (CML24) could affect autophagy progression and the resistance of darkness-induced starvation through interacting with ATG4 (Tsai et al., 2013). However, mechanistic details of the regulation between calcium signaling and autophagy in plants are not fully known. Additionally, early insightful studies have shown that cyclic nucleotide second messengers (cAMP and cGMP) regulate cellular autophagic capacity by directly affecting autophagy genes or indirect regulation in animals. The first insight comes from mammalian systems, where cAMP or dibutyryl cAMP injections produced a wave of autophagy in the rat liver (Shelburne et al., 1973). A number of subsequent studies have further explored the mechanism; for example, cyclic GMP-AMP (cGAMP) induced robust LC3 lipidation through WIPI2 (WD-repeat phosphatidylinositol-3-phosphate effector proteins) and ATG5-dependent pathways in human fibroblast cells (Gui et al., 2019).

## Transcriptional and Epigenetic Regulation of Autophagy

Recently, a growing body of research revealed that the transcriptional regulation of *ATG* genes is an important mechanism for autophagy to maintain cellular homeostasis under nutrient starvation and stress conditions. Furthermore, a growing number of studies also suggest that epigenetic changes, such as histone modification and DNA methylation, influence the expression of *ATG* genes and subsequent autophagic processes (Figure 2B).

### Transcription Factors

Transcription factors are important players controlling various processes of plant development and responses to different external stimuli. Increasing evidence in the last decade clearly indicates that nuclear transcriptional events play major roles in autophagy regulation under adverse environmental conditions in plants. Arabidopsis WRKY33 is the first reported TF that interacts with ATG18a and is required for resistance to necrotrophic pathogens (Lai et al., 2011). Likewise, its tomato homologs WRKY33a/b also play key roles in heat tolerance and regulation of stress-induced autophagy. Silencing *SlWRKY33s* reduced the expression of heat-induced *ATG* genes and the formation of autophagosomes (Zhou et al., 2014a). The first reported plant TF to transcriptionally regulate ATG genes was tomato HsfA1a, which directly binds to the promoters of *ATG10* and *ATG18f* and enhances their transcript levels under drought stress (Wang Y. et al., 2015). Subsequently, more TFs that regulate autophagy genes have been discovered, such as Arabidopsis elongated hypocotyl 5 (HY5), which directly binds to the promoters of *ATG5* and *ATG8e* to suppress their gene expression and thus negatively modulates autophagy (Yang et al., 2020b). Furthermore, key downstream signaling elements of phytohormones have also been reported to transcriptionally regulate autophagy genes. Tomato ERF5, a typical drought-responsive TF, is involved in ethylene-mediated autophagy through binding to the promoters of *ATG8d* and *ATG18h via* the DRE-binding site (ACCGAC) and promoting the expression of both genes (Zhu et al., 2018). BRs and their signaling element BZR1 can also transcriptionally upregulate *ATG* genes and the selective autophagy receptor *NBR1* and induce accumulation of NBR1 proteins and autophagosome formation in tomato under nitrogen starvation and chilling stress (Wang et al., 2019; Chi et al., 2020). These results indicate that TFs regulate *ATG* gene expression and promote autophagosome formation. Moreover, they are also involved in the regulation of selective autophagy receptors responsible for the recognition of damaged proteins. Furthermore, using a yeast one-hybrid library screening system, 225 TFs from 35 families were identified to bind to the promoters of *ATG8s*. These TFs are generally involved in plant development processes and environmental stress response (Wang P. et al., 2020). Whether more potential TFs are involved in plant autophagy signaling pathways remains to be demonstrated.

### DNA Methylation

DNA methylation is a major epigenetic modification that occurs in eukaryotes ranging from fungi to mammals. In plants, DNA methylation occurs in CG, CHG, and CHH (where H represents A, T, or C) sequence contexts and is mediated by DNA methyltransferases, like methyltransferase (MET), chromomethylase (CMT), and domain-rearranged methylase (DRM) (Qi et al., 2020). Several expression profiles have indicated that DNA methylation regulates autophagy genes in plants. For example, almost all *ATG* loci are enriched in different cytosine sequence contexts across gene regions in tomato (Zhong et al., 2013). Consistent with this, one genome-wide analysis of Arabidopsis DNA methylation also uncovered almost every *ATG* gene with a methylated modification (Zhong et al., 2015). Moreover, the methylation profiling of the DNA methyltransferase mutant *drm2* showed that the levels of methylated CG in *ATG6* and *ATG7* were lower than those in wild-type Arabidopsis (Zhong et al., 2015). Furthermore, the *ATG8f* promoter region was hypomethylated when evaluating global DNA methylation, and *ATG8f* expression was induced under TOR inhibition in Arabidopsis (Zhu et al., 2020). These studies allowed us to propose that DNA methylation plays a critical role in the regulation of autophagy in plants.

### Histone Modification

Histone modifications are essential transcriptional regulators that adjust chromatin structure and recruit histone modifiers. Histones contain five major subtypes (H1, H2A, H2B, H3, and H4) and include at least eight types of modifications: acetylation, methylation, phosphorylation, ubiquitylation, sumoylation, ADP ribosylation, deamination, and proline isomerization (Kouzarides, 2007). Accumulating evidence indicates that some histone modifications are related to the regulation of autophagy in animals. For instance, the methyltransferase enhancer of zeste homolog 2 (EZH2), which di- and trimethylates Lys27 of histone H3 (H3K27me2/3), can repress ATG5 and ATG7 protein levels in vascular smooth muscle cells. Moreover, inhibition or knockdown of *EZH2* induced the accumulation of ATG5 and ATG7 and autophagosome formation (Li et al., 2018). Plants generally possess histone modifications similar to those in animals. Arabidopsis histone deacetylase 9 (HDA9) can directly bind and repress the expression of the *ATG2*, *ATG9*, *ATG8e*, and *ATG13* genes. Moreover, HDA9 can be recruited by HY5 to *ATG5* and *ATG8e* loci to repress their expression through the deacetylation of H3K9 and H3K27 under nitrogen-sufficient or light conditions (Yang et al., 2020b). According to the enrichment analysis of essential histone markers on *ATG* genes in Arabidopsis, most *ATG* loci display high accumulation of the active markers H3K9/27/56Ac and H3K4/36me3, while only *ATG18e* shows enrichment of the repressive marker H3K27me3, which is closely related to the low expression of *ATG18e* in all Arabidopsis organs (Yang et al., 2020a).

### Noncoding RNAs

Noncoding RNAs (ncRNAs) have been shown to regulate a variety of cellular processes and functions by controlling gene expression. MicroRNAs (miRNAs) refer to a class of ncRNAs comprising 21–25 nucleotides that target multiple genes to regulate their expression. Long ncRNAs (lncRNAs) are noncoding transcripts of more than 200 nucleotides and have complex secondary structures to bind proteins, RNA, and DNA, thus endowing them with a variety of regulatory capabilities. Abundant studies in animals have shown that miRNAs and lncRNAs are broadly involved in the core pathways of autophagy, including vesicle nucleation, elongation, retrieval, and fusion. For instance, *MIR223* restrains autophagy and promotes central nervous system inflammation by targeting *ATG16L1*, and *MIR223* deficiency increases *ATG16L1* expression in murine cells (Li et al., 2019). In plants, one possible miRNA involved in the regulation of autophagy is *MIR447a.2*, which is highly expressed in Arabidopsis pollen and is predicted to target *ATG18h* (Borges et al., 2011). *MIR447a.2* could be induced by *Pseudomonas syringae* pv. *tomato* (*Pst* avrRpt2) infection in Arabidopsis, while the expression of its target gene *ATG18h* was reduced under *Pst* avrRpt2 treatment (Zhang et al., 2010). Compared with miRNAs, lncRNAs are larger and act through diverse sets of mechanisms to regulate autophagy in animals. For instance, the lncRNA *maternally expressed gene 3* (*MEG3*) upregulates *LC3* and *ATG3* expression levels, leading to autophagosome formation in epithelial ovarian cancer (Xiu et al., 2017). In plants, the roles of lncRNAs in the regulation of autophagy remain to be elucidated. Genome-wide analysis of 200 Arabidopsis transcriptome data sets successfully uncovered 6,480 lncRNAs, and the expression of 1,832 lncRNAs was significantly altered after drought, cold, high-salt, and ABA treatments (Matsui et al., 2008; Liu et al., 2012). Moreover, *ATG* genes were shown to be transcriptionally upregulated under these conditions (Liu et al., 2009; Chi et al., 2020; Wang M. et al., 2020). However, whether these *ATG* genes are modulated by lncRNAs remains unknown. Furthermore, the lncRNA-miRNA interaction also regulates autophagy at the molecular level in animals. *MIR188-3p* inhibits autophagy and cell death by targeting *ATG7*, while lncRNA *autophagy promoting factor* (*APF*) targets *MIR188-3p* and inhibits its activity; therefore, *APF* promotes autophagy signaling through targeting the *MIR188-3p*/*ATG7* axis in cardiomyocytes (Wang K. et al., 2015).

## Crosstalk Between Autophagy and the Ubiquitin–Proteasome System

Autophagy and the ubiquitin-26S proteasome pathway (UPS) constitute two major mechanisms of cellular protein degradation in eukaryotes, and they coordinately enable nutrient recycling, ensure cellular well-being and regulate growth (Pohl and Dikic, 2019). Unlike autophagy as an intracellular vesicle transport system, the UPS uses its own protease activity to degrade target proteins. UPS mediates the ubiquitination of target proteins by a three-step cascade of the E1 (activation), E2 (conjugation), and E3 (ligation) enzymes and then promotes the degradation of ubiquitinated proteins through the 26S proteasome in an ATP-dependent manner (Figure 2C). The 26S proteasome is a barrel-shaped organelle that is composed of the 20S core protease (CP) and lid 19S regulatory particles (RP) (Nam et al., 2017). It is now recognized that UPS normally aims to remove single, unfolded substrate polypeptides because the narrow entrance of the pore loops is only a 30- to 40-Å gap (Bard et al., 2018), while autophagy can degrade intact protein complexes, protein aggregates, or even organelles (Schreiber and Peter, 2014). Proteomic analyses have revealed that the ratio of protein degradation by proteasome or autophagy depends on cell types and states (Mathew et al., 2014; Braten et al., 2016). In addition, Arabidopsis autophagy receptor *nbr1* mutants and chaperone-associated E3 ubiquitin ligase *Hsc70-interacting protein* (*chip*) mutants both accumulated a large number of unfolded proteins under heat stress, and unfolded proteins were further increased in the *chip nbr1* double mutant (Zhou et al., 2014b), suggesting that both UPS and autophagy pathways collaboratively degrade aggregated proteins, and either pathway can be functionally compensated by the other when one is dysfunctional. Moreover, during plant aging, UPS mainly impacts the timing and onset of senescence, but autophagy is closely related to the degradation of bulk proteins during aging (Wang and Schippers, 2019).

Accumulating evidence has shown that crosstalk exists between autophagy and the proteasomal degradation pathway. Some autophagy components are directly regulated *via* ubiquitin-mediated UPS in plant cells. ATG6 can be ubiquitinated by the E3 ligases SINAT1 and SINAT2, leading to its degradation by the 26S proteasome in the presence of tumor necrosis factor receptor-associated factor 1a (TRAF1a) and TRAF1b in Arabidopsis (Qi et al., 2017). Moreover, inactivated proteasomes can be degraded by autophagy. For instance, the Arabidopsis 26S proteasome is the degradation substrate of ATG8-mediated autophagy (proteaphagy) under nitrogen starvation, and the RP non-ATPase subunit (RPN10) acts as a selective autophagy receptor in this process by targeting inactive 26S proteasomes and tethering them to autophagic vesicles. Furthermore, plant RPN10 serves as a dual receptor in both autophagy and the 26S proteasome pathway. RPN10 recognizes ubiquitylated targets when integrated into the 19S RP lid and has a specific UIM to bind to ATG8 docking sites (Marshall et al., 2015). Additionally, both Arabidopsis and tomato autophagy-deficient mutants hyperaccumulated ubiquitylated protein aggregates in response to heat and oxidative stresses, indicating that autophagy also recognizes ubiquitin and degrades ubiquitinylated substrates similar to the UPS (Zhou et al., 2013, 2014b). The mutual compensation mechanisms between autophagy and UPS are complex in plants, and further research is needed to integrate cellular protein quality control systems under different conditions.

## Agricultural Applications

**FIGURE 3:**
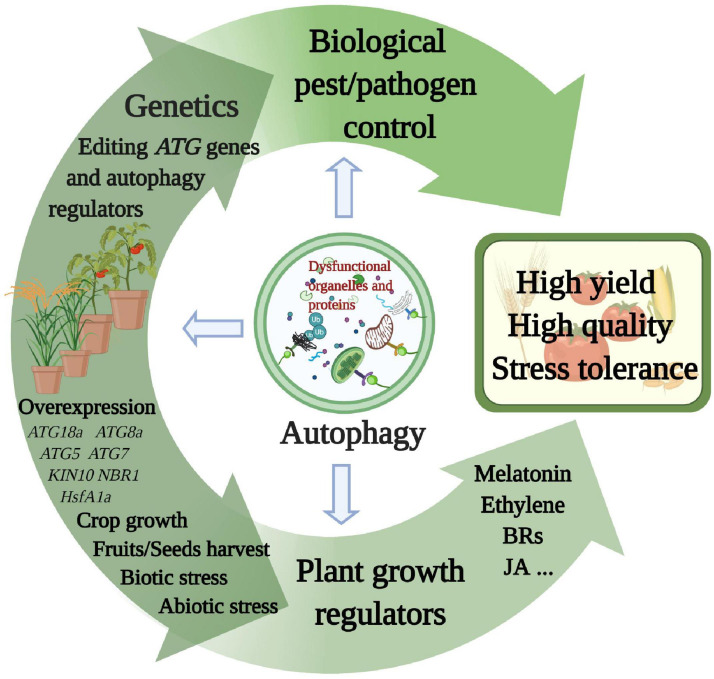
Multiple proposed agricultural applications related to autophagy. Each of the methods described in the main text explores a component of autophagy function. Superior yield, quality, and stress resistance characteristics will emerge in various crop species from autophagy applications *via* genetic research, plant growth regulators, and biological pest and pathogen control. This figure was created by BioRender (https://biorender.com/).

The functions of autophagy in growth, development, and stress responses have been deciphered in various crop species. Therefore, how to use autophagy to improve agricultural benefits, such as high yield, quality, and multifaceted resistance, is an important research direction. Here, we highlight the roles of autophagy in crop growth, yield, and stress tolerance and discuss future research directions for potential applications in agriculture (Figure 3).

### Utilizing Plant Growth Regulators and Biological Pest/Pathogen Control

As discussed above, some plant growth regulators can regulate autophagy; thus, the application of growth regulators may be an effective way to activate autophagy for agricultural benefits. For instance, elevation of both exogenous and endogenous melatonin results in enhanced thermotolerance in tomato mediated by *ATG* expression and autophagy, which are related to the degradation of aggregated proteins under heat stress (Xu et al., 2016). Exogenous BRs can be used to increase crop resistance to nitrogen starvation and chilling stress through BZR1-mediated autophagy (Wang et al., 2019; Chi et al., 2020). Thus, melatonin and BRs are possibly utilized as plant growth regulators to increase crop resistance through autophagy signaling. Ethylene has also been suggested to mediate autophagy, contributing to enhanced survival during flooding, hypoxia, and reoxygenation stress through the induction of *ATG* genes and ROS levels in soybean and tomato (Hartman et al., 2019). Considering its function in fruit ripening and leaf senescence, the manipulation of ethylene might be an effective means to regulate plant growth and fruit quality *via* autophagy. Moreover, plant resistance against necrotrophic phytopathogens is mediated by autophagy *via* modulation of hormone homeostasis. For example, autophagy apparently plays a positive role in the induction of jasmonic acid (JA)-regulated *plant defensin 1.2* (*PDF1.2*) expression as a defense against *Botrytis cinerea* in Arabidopsis (Lai et al., 2011). In addition, we can exploit the corresponding biological pesticides to manipulate autophagic activity in pathogens and pests. For instance, *Tomato yellow leaf curl virus* (TYLCV) is a whitefly-transmitted geminivirus that causes severe yield losses in tomato production. Activating autophagy in whitefly inhibits the transmission efficiency of TYLCV by reducing the amount of viral coat protein and genomic DNA transmitted to tomato plants. Feeding with rapamycin activates whitefly autophagy to prevent TYLCV transmission to solanaceous plants (Wang et al., 2016). Interestingly, autophagy induced by different pathogens in insect vectors causes different results; for example, the *Rice gall dwarf virus* (RGDV)-induced autophagy pathway promotes viral replication in the leafhopper *Recilia dorsalis*, causing severe viral infection and transmission to rice plants. Moreover, leafhopper-borne viral spread was decreased by the autophagy inhibitor 3MA (Chen Y. et al., 2017). Thus, by exploring the function and the regulation of autophagy in insect-borne pathogens, we can exploit pesticides that can activate/inhibit the autophagy pathway in insect vectors and block vector-borne plant viruses. With ongoing autophagy research, plant growth regulators and pesticides will be developed in agricultural production based on autophagy signaling.

### Exploring Potential Applications of Functional Genes

The evolution and practical breeding of crops essentially depend on genetic variation. With the development of genomics and genome-editing techniques, we are able to select excellent agronomic traits and increase yields by editing *ATG* genes and autophagy regulators. For example, overexpression of foxtail millet *SiATG8a* in Arabidopsis and rice *OsATG8a* conferred tolerance to nitrogen starvation, with an increase in root and leaf areas and increased nitrogen absorption (Izumi et al., 2015; Li et al., 2015). Overexpression of apple *MdATG18a* also improved nitrogen absorption by upregulating nitrate uptake genes and the accumulation of anthocyanins (Sun et al., 2018), indicating that the application of *ATG* genes contributes to crop adaptability to low-nitrogen environments and improves crop growth. Moreover, autophagy also affects pollen growth, development, yield, and fruit ripening. For example, rice mutants defective in autophagy showed sporophytic male sterility and immature pollen (Kurusu and Kuchitsu, 2017). Ripened pepper fruits exhibited increases in the expression of *ATG4*, *ATG8a*, and *ATG9* (Lopez-Vidal et al., 2020), and postharvest fruit senescence of *Ziziphus jujube* was delayed by the inhibition of autophagy (Deng et al., 2019). Constitutive overexpression of *ATG5* or *ATG7* increased seed yields and the levels of fatty acids in Arabidopsis seeds (Minina et al., 2018), suggesting that upregulated autophagy has a positive effect on increasing crop fitness and oil accumulation in the breeding of high-yield oil crops (Ortiz et al., 2020). However, these overexpressing transgenic plants are generated by increasing protein expression from cloned transgenes with special enhancers. Considering the biosafety of agricultural products, transgene-free genome editing might be a better way to expedite crop improvement by enhancing *ATG* gene expression. Manipulation of upstream open reading frames (uORFs) by genome editing can fine-tune mRNA translation and thereby increase the amounts of protein synthesized (Zhang et al., 2018).

Autophagy is widely regarded to enhance stress resistance. Overexpression of *ATG8* conferred tolerance to low-nitrate conditions and led to an increase in yield and nitrogen remobilization efficiency in Arabidopsis (Chen et al., 2019). Overexpression of *ATG5* and *ATG7* increased ATG8 protein lipidation and autophagic flux, thereby exhibiting increased resistance to necrotrophic pathogens and oxidative stress in Arabidopsis (Minina et al., 2018). Overexpression of *Joka2* (homolog of *NBR1*) significantly restricted the size of disease lesions caused by *Phytophthora infestans* in potato, but virus-induced gene silencing of *Joka2* resulted in increased disease lesions, indicating that *Joka2*-mediated selective autophagy contributes to defense against *P. infestans* (Dagdas et al., 2016). Therefore, manipulation of *ATG* transcriptional changes by genetic stimulation seems to be an effective approach for enhancing plant resistance.

In addition to directly focusing on *ATG* genes in various stages of autophagy, multiplex gene editing, knockouts, and regulation of gene transcription can be used to regulate upstream signaling pathways of autophagy. For example, overexpressing the tomato TF *HsfA1a*, which activates *ATG* genes, would enhance plant drought tolerance (Wang Y. et al., 2015). TOR and SnRK1 are essential upstream regulators of autophagy. Downregulation of *TOR* expression or kinase activity led to constitutive activation of autophagy, while the overexpression of *TOR* was sufficient to block starvation-, salt-, and drought-induced autophagy in Arabidopsis (Liu and Bassham, 2010; Pu et al., 2017a). Arabidopsis *KIN10*-overexpressing lines exhibited enhanced tolerance to hypoxia with increasing autophagy (Chen L. et al., 2017; Janse van Rensburg et al., 2019).

## Conclusion and Future Perspectives

The core mechanisms of autophagy are conserved in all eukaryotes and have been well-studied in plants. However, the functions and regulatory networks of autophagy are still not fully understood. First, although many *ATG* genes have been identified, their multiple functions beyond self-eating are still unknown, and some ATG proteins may play multiple roles and have pleiotropic effects during numerous processes or pathways. For instance, ATG1 is an essential protein for the formation of autophagy vesicles and can be phosphorylated by the upstream kinase TOR; thus, is there a possibility that ATG1, as a kinase, regulates other signaling pathways besides autophagy? ATG10 acts as an E2-like enzyme to help ATG5 conjugate with ATG12; is there a possibility that ATG10, as an E2-like enzyme, has other substrates besides autophagy? Therefore, the multiple functions of ATG are interesting research topics that warrant further investigation in plants. Second, the regulatory networks of autophagy are intricate, and different levels of autophagy regulation might play complex and ambiguous roles. Different phytohormones are mutually antagonistic and synergistic during plant growth and development or under stress. Phytohormones regulate autophagy, and in turn, autophagy feedback influences the balance of phytohormones. *ATG*-overexpressing and *atg* mutant plants can both exhibit high levels of the same phytohormones, suggesting that crosstalk interactions between hormonal signals and autophagy are more complicated. Third, the role of autophagy remains to be further explored, and more precise approaches are needed to provide clearer insights into the agricultural applications of autophagy, such as artificial evolution of critical autophagy genes by base editing. Overall, in the coming years, more interesting and fundamental research will likely emerge to answer existing questions regarding plant autophagy and will shine light on agricultural applications.

## Author Contributions

JZ planned the review manuscript. J-JC and C-XL analyzed the data. J-JC, JZ, and S-JS wrote the manuscript. All authors contributed to the article and approved the submitted version.

## Conflict of Interest

The authors declare that the research was conducted in the absence of any commercial or financial relationships that could be construed as a potential conflict of interest.

## Supplementary Material

Supplementary Figure 1 DNA methylation in plants.

Supplementary Table 1 DNA methylation of Arabidopsis ATG genes.

## References

[B1] Abdrakhmanov A. Gogvadze V. Zhivotovsky B. (2020). To eat or to die: deciphering selective forms of autophagy. *Trends Biochem. Sci.* 45 347–364. 10.1016/j.tibs.2019.11.006 32044127

[B2] Acheampong A. K. Shanks C. Cheng C. Y. Schaller G. E. Dagdas Y. Kieber J. J. (2020). EXO70D isoforms mediate selective autophagic degradation of type-A ARR proteins to regulate cytokinin sensitivity. *Proc. Natl. Acad. Sci. U.S.A.* 117 27034–27043. 10.1073/pnas.2013161117 33051300 PMC7604425

[B3] Antonioli M. Di Rienzo M. Piacentini M. Fimia G. M. (2017). Emerging mechanisms in initiating and terminating autophagy. *Trends Biochem. Sci.* 42 28–41. 10.1016/j.tibs.2016.09.008 27765496

[B4] Bao Y. Bassham D. C. (2020). COST1 balances plant growth and stress tolerance via attenuation of autophagy. *Autophagy* 16 1157–1158. 10.1080/15548627.2020.1752981 32268813 PMC7469670

[B5] Bao Y. Pu Y. Yu X. Gregory B. D. Srivastava R. Howell S. H. (2018). IRE1B degrades RNAs encoding proteins that interfere with the induction of autophagy by ER stress in *Arabidopsis thaliana*. *Autophagy* 14 1562–1573. 10.1080/15548627.2018.1462426 29940799 PMC6135571

[B6] Bard J. A. M. Goodall E. A. Greene E. R. Jonsson E. Dong K. C. Martin A. (2018). Structure and function of the 26S proteasome. *Annu. Rev. Biochem.* 87 697–724. 10.1146/annurev-biochem-062917-011931 29652515 PMC6422034

[B7] Bassham D. C. Laporte M. Marty F. Moriyasu Y. Ohsumi Y. Olsen L. J. (2006). Autophagy in development and stress responses of plants. *Autophagy* 2 2–11. 10.4161/auto.2092 16874030

[B8] Belda-Palazon B. Adamo M. Valerio C. Ferreira L. J. Confraria A. Reis-Barata D. (2020). A dual function of SnRK2 kinases in the regulation of SnRK1 and plant growth. *Nat. Plants* 6 1345–1353. 10.1038/s41477-020-00778-w 33077877

[B9] Borek S. Stefaniak S. Sliwinski J. Garnczarska M. Pietrowska-Borek M. (2019). Autophagic machinery of plant peroxisomes. *Int. J. Mol. Sci.* 20 4754. 10.3390/ijms20194754 31557865 PMC6802006

[B10] Borges F. Pereira P. A. Slotkin R. K. Martienssen R. A. Becker J. D. (2011). MicroRNA activity in the *Arabidopsis male* germline. *J. Exp. Bot.* 62 1611–1620. 10.1093/jxb/erq452 21357774 PMC5536363

[B11] Braten O. Livneh I. Ziv T. Admon A. Kehat I. Caspi L. H. (2016). Numerous proteins with unique characteristics are degraded by the 26S proteasome following monoubiquitination. *Proc. Natl. Acad. Sci. U.S.A.* 113 e4639–e4647. 10.1073/pnas.1608644113 27385826 PMC4987823

[B12] Chanoca A. Kovinich N. Burkel B. Stecha S. Bohorquez-Restrepo A. Ueda T. (2015). Anthocyanin vacuolar inclusions form by a microautophagy mechanism. *Plant Cell* 27 2545–2559. 10.1105/tpc.15.00589 26342015 PMC4815043

[B13] Chen L. Su Z. Z. Huang L. Xia F. N. Qi H. Xie L. J. (2017). The AMP-activated protein kinase KIN10 is involved in the regulation of autophagy in *Arabidopsis*. *Front. Plant Sci.* 8:1201. 10.3389/fpls.2017.01201 28740502 PMC5502289

[B14] Chen Q. Soulay F. Saudemont B. Elmayan T. Marmagne A. Masclaux-Daubresse C. (2019). Overexpression of ATG8 in *Arabidopsis* stimulates autophagic activity and increases nitrogen remobilization efficiency and grain filling. *Plant Cell Physiol.* 60 343–352. 10.1093/pcp/pcy214 30407574

[B15] Chen Y. Chen Q. Li M. Mao Q. Chen H. Wu W. (2017). Autophagy pathway induced by a plant virus facilitates viral spread and transmission by its insect vector. *PLoS Pathog.* 13:e1006727. 10.1371/journal.ppat.1006727 29125860 PMC5708841

[B16] Chi C. Li X. Fang P. Xia X. Shi K. Zhou Y. (2020). Brassinosteroids act as a positive regulator of NBR1-dependent selective autophagy in response to chilling stress in tomato. *J. Exp. Bot.* 71 1092–1106. 10.1093/jxb/erz466 31639824

[B17] Dagdas Y. F. Belhaj K. Maqbool A. Chaparro-Garcia A. Pandey P. Petre B. (2016). An effector of the Irish potato famine pathogen antagonizes a host autophagy cargo receptor. *eLife* 5 e10856. 10.7554/eLife.10856 26765567 PMC4775223

[B18] Deng B. Guo M. Liu H. Tian S. Zhao X. (2019). Inhibition of autophagy by hydroxychloroquine enhances antioxidant nutrients and delays postharvest fruit senescence of *Ziziphus jujuba*. *Food Chem.* 296 56–62. 10.1016/j.foodchem.2019.05.189 31202306

[B19] Furukawa K. Innokentev A. Kanki T. (2019). Regulatory mechanisms of mitochondrial autophagy: lessons from yeast. *Front. Plant Sci.* 10:1479. 10.3389/fpls.2019.01479 31803214 PMC6872543

[B20] Galluzzi L. Baehrecke E. H. Ballabio A. Boya P. Pedro J. M. B.-S. Cecconi F. (2017). Molecular definitions of autophagy and related processes. *EMBO J.* 36 1811–1836. 10.15252/embj.201796697 28596378 PMC5494474

[B21] Gordon P. B. Holen I. Fosse M. Rotnes J. S. Seglen P. O. (1993). Dependence of hepatocytic autophagy on intracellularly sequestered calcium. *J. Biol. Chem.* 268 26107–26112. 10.1016/s0021-9258(19)74287-2 8253727

[B22] Gui X. Yang H. Li T. Tan X. Shi P. Li M. (2019). Autophagy induction via STING trafficking is a primordial function of the cGAS pathway. *Nature* 567 262–266. 10.1038/s41586-019-1006-9 30842662 PMC9417302

[B23] Hartman S. Sasidharan R. Voesenek L. (2019). The role of ethylene in metabolic acclimations to low oxygen. *New Phytol.* 229 64–70. 10.1111/nph.16378 31856295 PMC7754284

[B24] Huang L. Yu L. J. Zhang X. Fan B. Wang F. Z. Dai Y. S. (2019). Autophagy regulates glucose-mediated root meristem activity by modulating ROS production in *Arabidopsis*. *Autophagy* 15 407–422. 10.1080/15548627.2018.1520547 30208757 PMC6351127

[B25] Huang X. Zheng C. Liu F. Yang C. Zheng P. Lu X. (2019). Genetic analyses of the Arabidopsis ATG1 kinase complex reveal both kinase-dependent and independent autophagic routes during fixed-carbon starvation. *Plant Cell* 31 2973–2995. 10.1105/tpc.19.00066 31615848 PMC6925010

[B26] Hurley J. H. Young L. N. (2017). Mechanisms of autophagy initiation. *Annu. Rev. Biochem.* 86 225–244. 10.1146/annurev-biochem-061516-044820 28301741 PMC5604869

[B27] Izumi M. Hidema J. Wada S. Kondo E. Kurusu T. Kuchitsu K. (2015). Establishment of monitoring methods for autophagy in rice reveals autophagic recycling of chloroplasts and root plastids during energy limitation. *Plant Physiol.* 167 1307–1320. 10.1104/pp.114.254078 25717038 PMC4378162

[B28] Janse van Rensburg H. C. Van den Ende W. Signorelli S. (2019). Autophagy in plants: both a puppet and a puppet master of sugars. *Front. Plant Sci.* 10:14. 10.3389/fpls.2019.00014 30723485 PMC6349728

[B29] Johansen T. Lamark T. (2020). Selective autophagy: ATG8 family proteins, LIR motifs and cargo receptors. *J. Mol. Biol.* 432 80–103. 10.1016/j.jmb.2019.07.016 31310766

[B30] Kamada Y. Yoshino K. Kondo C. Kawamata T. Oshiro N. Yonezawa K. (2010). TOR directly controls the ATG1 kinase complex to regulate autophagy. *Mol. Cell Biol.* 30 1049–1058. 10.1128/MCB.01344-09 19995911 PMC2815578

[B31] Kim J. Kundu M. Viollet B. Guan K. L. (2011). AMPK and mTOR regulate autophagy through direct phosphorylation of Ulk1. *Nat. Cell Biol.* 13 132–141. 10.1038/ncb2152 21258367 PMC3987946

[B32] Kouzarides T. (2007). Chromatin modifications and their function. *Cell* 128 693–705. 10.1016/j.cell.2007.02.005 17320507

[B33] Kurusu T. Koyano T. Kitahata N. Kojima M. Hanamata S. Sakakibara H. (2017). Autophagy-mediated regulation of phytohormone metabolism during rice anther development. *Plant Signal. Behav.* 12 e1365211. 10.1080/15592324.2017.1365211 28873038 PMC5640179

[B34] Kurusu T. Kuchitsu K. (2017). Autophagy, programmed cell death and reactive oxygen species in sexual reproduction in plants. *J. Plant Res.* 130 491–499. 10.1007/s10265-017-0934-4 28364377

[B35] Kwon S. I. Cho H. J. Jung J. H. Yoshimoto K. Shirasu K. Park O. K. (2010). The Rab GTPase RabG3b functions in autophagy and contributes to tracheary element differentiation in *Arabidopsis*. *Plant J.* 64 151–164. 10.1111/j.1365-313X.2010.04315.x 20659276

[B36] Lai Z. Wang F. Zheng Z. Fan B. Chen Z. (2011). A critical role of autophagy in plant resistance to necrotrophic fungal pathogens. *Plant J.* 66 953–968. 10.1111/j.1365-313X.2011.04553.x 21395886

[B37] Laureano-Marin A. M. Aroca A. Perez-Perez M. E. Yruela I. Jurado-Flores A. Moreno I. (2020). Abscisic acid-triggered persulfidation of cysteine protease ATG4 mediates regulation of autophagy by sulfide. *Plant Cell* 32 3902–3920. 10.1105/tpc.20.00766 33037147 PMC7721334

[B38] Leary A. Y. Savage Z. Tumtas Y. Bozkurt T. O. (2019). Contrasting and emerging roles of autophagy in plant immunity. *Curr. Opin. Plant Biol.* 52 46–53. 10.1016/j.pbi.2019.07.002 31442734

[B39] Li F. Vierstra R. D. (2012). Regulator and substrate: dual roles for the ATG1-ATG13 kinase complex during autophagic recycling in *Arabidopsis*. *Autophagy* 8 982–984. 10.4161/auto.20240 22714291 PMC3427266

[B40] Li R. Yi X. Wei X. Huo B. Guo X. Cheng C. (2018). EZH2 inhibits autophagic cell death of aortic vascular smooth muscle cells to affect aortic dissection. *Cell Death Dis.* 9 180. 10.1038/s41419-017-0213-2 29416002 PMC5833461

[B41] Li W. W. Chen M. Zhong L. Liu J. M. Xu Z. S. Li L. C. (2015). Overexpression of the autophagy-related gene SiATG8a from foxtail millet (*Setaria italica* L.) confers tolerance to both nitrogen starvation and drought stress in *Arabidopsis*. *Biochem. Biophys. Res. Commun.* 468 800–806. 10.1016/j.bbrc.2015.11.035 26577407

[B42] Li Y. Zhou D. Ren Y. Zhang Z. Guo X. Ma M. (2019). Mir223 restrains autophagy and promotes CNS inflammation by targeting ATG16L1. *Autophagy* 15 478–492. 10.1080/15548627.2018.1522467 30208760 PMC6351131

[B43] Liu F. Hu W. Li F. Marshall R. S. Zarza X. Munnik T. (2020). AUTOPHAGY-RELATED14 and its associated phosphatidylinositol 3-kinase complex promotes autophagy in *Arabidopsis*. *Plant Cell* 32 3939–3960. 10.1105/tpc.20.00285 33004618 PMC7721316

[B44] Liu J. Jung C. Xu J. Wang H. Deng S. Bernad L. (2012). Genome-wide analysis uncovers regulation of long intergenic noncoding RNAs in *Arabidopsis*. *Plant Cell* 24 4333–4345. 10.1105/tpc.112.102855 23136377 PMC3531837

[B45] Liu Y. Bassham D. C. (2010). TOR is a negative regulator of autophagy in *Arabidopsis thaliana*. *PLoS One* 5:e11883. 10.1371/journal.pone.0011883 20686696 PMC2912371

[B46] Liu Y. Bassham D. C. (2012). Autophagy: pathways for self-eating in plant cells. *Annu. Rev. Plant Biol.* 63 215–237. 10.1146/annurev-arplant-042811-105441 22242963

[B47] Liu Y. Xiong Y. Bassham D. C. (2009). Autophagy is required for tolerance of drought and salt stress in plants. *Autophagy* 5 954–963. 10.4161/auto.5.7.9290 19587533

[B48] Lopez-Vidal O. Olmedilla A. Sandalio L. M. Sevilla F. Jimenez A. (2020). Is autophagy involved in pepper fruit ripening? *Cells* 9 106. 10.3390/cells9010106 31906273 PMC7016703

[B49] Marshall R. S. Hua Z. Mali S. McLoughlin F. Vierstra R. D. (2019). ATG8-binding UIM proteins define a new class of autophagy adaptors and receptors. *Cell* 177 766–781. 10.1016/j.cell.2019.02.009 30955882 PMC6810650

[B50] Marshall R. S. Li F. Gemperline D. C. Book A. J. Vierstra R. D. (2015). Autophagic degradation of the 26S proteasome is mediated by the dual ATG8/ubiquitin receptor RPN10 in *Arabidopsis*. *Mol. Cell* 58 1053–1066. 10.1016/j.molcel.2015.04.023 26004230 PMC4903074

[B51] Marshall R. S. Vierstra R. D. (2018). Autophagy: the master of bulk and selective recycling. *Annu. Rev. Plant Biol.* 69 173–208. 10.1146/annurev-arplant-042817-040606 29539270

[B52] Mathew R. Khor S. Hackett S. R. Rabinowitz J. D. Perlman D. H. White E. (2014). Functional role of autophagy-mediated proteome remodeling in cell survival signaling and innate immunity. *Mol. Cell* 55 916–930. 10.1016/j.molcel.2014.07.019 25175026 PMC4169768

[B53] Matsui A. Ishida J. Morosawa T. Mochizuki Y. Kaminuma E. Endo T. A. (2008). Arabidopsis transcriptome analysis under drought, cold, high-salinity and ABA treatment conditions using a tiling array. *Plant Cell Physiol.* 49 1135–1149. 10.1093/pcp/pcn101 18625610

[B54] Medeiros D. B. Barros J. A. S. Fernie A. R. Araujo W. L. (2020). Eating away at ROS to regulate stomatal opening. *Trends Plant Sci.* 25 220–223. 10.1016/j.tplants.2019.12.023 31932167

[B55] Michaeli S. Galili G. Genschik P. Fernie A. R. Avin-Wittenberg T. (2016). Autophagy in plants–What’s new on the menu? *Trends Plant Sci.* 21 134–144. 10.1016/j.tplants.2015.10.008 26598298

[B56] Minina E. A. Moschou P. N. Vetukuri R. R. Sanchez-Vera V. Cardoso C. Liu Q. (2018). Transcriptional stimulation of rate-limiting components of the autophagic pathway improves plant fitness. *J. Exp. Bot.* 69 1415–1432. 10.1093/jxb/ery010 29365132 PMC6019011

[B57] Mittler R. (2017). ROS are good. *Trends Plant Sci.* 22 11–19. 10.1016/j.tplants.2016.08.002 27666517

[B58] Munch D. Rodriguez E. Bressendorff S. Park O. K. Hofius D. Petersen M. (2014). Autophagy deficiency leads to accumulation of ubiquitinated proteins, ER stress, and cell death in *Arabidopsis*. *Autophagy* 10 1579–1587. 10.4161/auto.29406 25046116 PMC4206536

[B59] Nam T. Han J. H. Devkota S. Lee H. W. (2017). Emerging paradigm of crosstalk between autophagy and the ubiquitin-proteasome system. *Mol. Cells* 40 897–905. 10.14348/molcells.2017.0226 29237114 PMC5750708

[B60] Nolan T. M. Brennan B. Yang M. Chen J. Zhang M. Li Z. (2017). Selective autophagy of BES1 mediated by DSK2 balances plant growth and survival. *Dev. Cell* 41 e37. 10.1016/j.devcel.2017.03.013 28399398 PMC5720862

[B61] Nukarinen E. Nagele T. Pedrotti L. Wurzinger B. Mair A. Landgraf R. (2016). Quantitative phosphoproteomics reveals the role of the AMPK plant ortholog SnRK1 as a metabolic master regulator under energy deprivation. *Sci. Rep.* 6 31697. 10.1038/srep31697 27545962 PMC4992866

[B62] Ortiz R. Geleta M. Gustafsson C. Lager I. Hofvander P. Lofstedt C. (2020). Oil crops for the future. *Curr. Opin. Plant Biol.* 56 181–189. 10.1016/j.pbi.2019.12.003 31982290

[B63] Perez-Perez M. E. Lemaire S. D. Crespo J. L. (2016). Control of autophagy in Chlamydomonas is mediated through redox-dependent inactivation of the ATG4 protease. *Plant Physiol.* 172 2219–2234. 10.1104/pp.16.01582 27756818 PMC5129734

[B64] Pohl C. Dikic I. (2019). Cellular quality control by the ubiquitin-proteasome system and autophagy. *Science* 366 818–822. 10.1126/science.aax3769 31727826

[B65] Pu Y. Luo X. Bassham D. C. (2017a). TOR-dependent and -independent pathways regulate autophagy in *Arabidopsis thaliana*. *Front. Plant Sci.* 8:1204. 10.3389/fpls.2017.01204 28744293 PMC5504165

[B66] Pu Y. Soto-Burgos J. Bassham D. C. (2017b). Regulation of autophagy through SnRK1 and TOR signaling pathways. *Plant Signal. Behav.* 12 e1395128. 10.1080/15592324.2017.1395128 29058995 PMC5792129

[B67] Qi H. Xia F. N. Xiao S. (2020). Autophagy in plants: physiological roles and post-translational regulation. *J. Integr. Plant Biol.* 10.1111/jipb.12941 [Epub ahead of print]. 32324339

[B68] Qi H. Xia F. N. Xie L. J. Yu L. J. Chen Q. F. Zhuang X. H. (2017). TRAF family proteins regulate autophagy dynamics by modulating AUTOPHAGY PROTEIN6 stability in *Arabidopsis*. *Plant Cell* 29 890–911. 10.1105/tpc.17.00056 28351989 PMC5435438

[B69] Rodriguez M. Parola R. Andreola S. Pereyra C. Martinez-Noel G. (2019). TOR and SnRK1 signaling pathways in plant response to abiotic stresses: do they always act according to the “yin-yang” model? *Plant Sci.* 288 110220. 10.1016/j.plantsci.2019.110220 31521220

[B70] Schreiber A. Peter M. (2014). Substrate recognition in selective autophagy and the ubiquitin-proteasome system. *Biochim. Biophys. Acta* 1843 163–181. 10.1016/j.bbamcr.2013.03.019 23545414

[B71] Shelburne J. D. Arstila A. U. Trump B. F. (1973). Studies on cellular autophagocytosis: cyclic Amp-stimulated and dibutyryl cyclic Amp-stimulated autophagy in rat liver. *Am. J. Pathol.* 72 521–539. 4125701 PMC1904032

[B72] Shibata M. Oikawa K. Yoshimoto K. Kondo M. Mano S. Yamada K. (2013). Highly oxidized peroxisomes are selectively degraded via autophagy in *Arabidopsis*. *Plant Cell* 25 4967–4983. 10.1105/tpc.113.116947 24368788 PMC3903999

[B73] Slavikova S. Ufaz S. Avin-Wittenberg T. Levanony H. Galili G. (2008). An autophagy-associated ATG8 protein is involved in the responses of Arabidopsis seedlings to hormonal controls and abiotic stresses. *J. Exp. Bot.* 59 4029–4043. 10.1093/jxb/ern244 18836138 PMC2576633

[B74] Su T. Li X. Yang M. Shao Q. Zhao Y. Ma C. (2020). Autophagy: an intracellular degradation pathway regulating plant survival and stress response. *Front. Plant Sci.* 11:164. 10.3389/fpls.2020.00164 32184795 PMC7058704

[B75] Sun X. Jia X. Huo L. Che R. Gong X. Wang P. (2018). MdATG18a overexpression improves tolerance to nitrogen deficiency and regulates anthocyanin accumulation through increased autophagy in transgenic apple. *Plant Cell Environ.* 41 469–480. 10.1111/pce.13110 29210078

[B76] Suttangkakul A. Li F. Chung T. Vierstra R. D. (2011). The ATG1/ATG13 protein kinase complex is both a regulator and a target of autophagic recycling in *Arabidopsis*. *Plant Cell* 23 3761–3779. 10.1105/tpc.111.090993 21984698 PMC3229148

[B77] Tang J. Bassham D. C. (2018). Autophagy in crop plants: what’s new beyond *Arabidopsis*? *Open Biol.* 8 180162. 10.1098/rsob.180162 30518637 PMC6303781

[B78] Tsai Y. C. Koo Y. Delk N. A. Gehl B. Braam J. (2013). Calmodulin-related CML24 interacts with ATG4b and affects autophagy progression in *Arabidopsis*. *Plant J.* 73 325–335. 10.1111/tpj.12043 23039100

[B79] Turco E. Witt M. Abert C. Bock-Bierbaum T. Su M. Y. Trapannone R. (2019). FIP200 claw domain binding to p62 promotes autophagosome formation at ubiquitin condensates. *Mol. Cell* 74 330–346. 10.1016/j.molcel.2019.01.035 30853400 PMC6477179

[B80] van Doorn W. G. Woltering E. J. (2005). Many ways to exit? Cell death categories in plants. *Trends Plant Sci.* 10 117–122. 10.1016/j.tplants.2005.01.006 15749469

[B81] Vanhee C. Batoko H. (2011). Autophagy involvement in responses to abscisic acid by plant cells. *Autophagy* 7 655–656. 10.4161/auto.7.6.15307 21460617

[B82] Vargas J. N. S. Wang C. Bunker E. Hao L. Maric D. Schiavo G. (2019). Spatiotemporal control of ULK1 activation by NDP52 and TBK1 during selective autophagy. *Mol. Cell* 74 347–362. 10.1016/j.molcel.2019.02.010 30853401 PMC6642318

[B83] Wang H. Schippers J. H. M. (2019). The role and regulation of autophagy and the proteasome during aging and senescence in plants. *Genes* 10 267. 10.3390/genes10040267 30987024 PMC6523301

[B84] Wang K. Liu C. Y. Zhou L. Y. Wang J. X. Wang M. Zhao B. (2015). APF lncRNA regulates autophagy and myocardial infarction by targeting miR-188-3p. *Nat. Commun.* 6 6779. 10.1038/ncomms7779 25858075

[B85] Wang L. L. Wang X. R. Wei X. M. Huang H. Wu J. X. Chen X. X. (2016). The autophagy pathway participates in resistance to tomato yellow leaf curl virus infection in whiteflies. *Autophagy* 12 1560–1574. 10.1080/15548627.2016.1192749 27310765 PMC5082773

[B86] Wang M. Li X. Luo S. Fan B. Zhu C. Chen Z. (2020). Coordination and crosstalk between autophagosome and multivesicular body pathways in plant stress responses. *Cells* 9 119. 10.3390/cells9010119 31947769 PMC7017292

[B87] Wang P. Nolan T. M. Yin Y. Bassham D. C. (2020). Identification of transcription factors that regulate ATG8 expression and autophagy in *Arabidopsis*. *Autophagy* 16 123–139. 10.1080/15548627.2019.1598753 30909785 PMC6984607

[B88] Wang P. Zhao Y. Li Z. Hsu C. C. Liu X. Fu L. (2018). Reciprocal eregulation of the TOR kinase and ABA receptor balances plant growth and stress response. *Mol. Cell* 69 100–112. 10.1016/j.molcel.2017.12.002 29290610 PMC5772982

[B89] Wang Y. Cai S. Y. Yin L. L. Shi K. Xia X. Zhou Y. H. (2015). Tomato HsfA1a plays a critical role in plant drought tolerance by activating ATG genes and inducing autophagy. *Autophagy* 11 2033–2047. 10.1080/15548627.2015.1098798 26649940 PMC4824577

[B90] Wang Y. Cao J. J. Wang K. X. Xia X. J. Shi K. Zhou Y. H. (2019). BZR1 mediates brassinosteroid-induced autophagy and nitrogen starvation in tomato. *Plant Physiol.* 179 671–685. 10.1104/pp.18.01028 30482787 PMC6426427

[B91] Witters L. A. Kemp B. E. Means A. R. (2006). Chutes and ladders: the search for protein kinases that act on AMPK. *Trends Biochem. Sci.* 31 13–16. 10.1016/j.tibs.2005.11.009 16356723

[B92] Woo J. Park E. Dinesh-Kumar S. P. (2014). Differential processing of *Arabidopsis ubiquitin*-like ATG8 autophagy proteins by ATG4 cysteine proteases. *Proc. Natl. Acad. Sci. U.S.A.* 111 863–868. 10.1073/pnas.1318207111 24379391 PMC3896200

[B93] Wurzinger B. Mair A. Fischer-Schrader K. Nukarinen E. Roustan V. Weckwerth W. (2017). Redox state-dependent modulation of plant SnRK1 kinase activity differs from AMPK regulation in animals. *FEBS Lett.* 591 3625–3636. 10.1002/1873-3468.12852 28940407 PMC5698759

[B94] Xiong Y. Contento A. L. Nguyen P. Q. Bassham D. C. (2007). Degradation of oxidized proteins by autophagy during oxidative stress in *Arabidopsis*. *Plant Physiol.* 143 291–299. 10.1104/pp.106.092106 17098847 PMC1761971

[B95] Xiu Y. L. Sun K. X. Chen X. Chen S. Zhao Y. Guo Q. G. (2017). Upregulation of the lncRNA Meg3 induces autophagy to inhibit tumorigenesis and progression of epithelial ovarian carcinoma by regulating activity of ATG3. *Oncotarget* 8 31714–31725. 10.18632/oncotarget.15955 28423647 PMC5458242

[B96] Xu W. Cai S. Y. Zhang Y. Wang Y. Ahammed G. J. Xia X. J. (2016). Melatonin enhances thermotolerance by promoting cellular protein protection in tomato plants. *J. Pineal Res.* 61 457–469. 10.1111/jpi.12359 27484733

[B97] Yamauchi S. Mano S. Oikawa K. Hikino K. Teshima K. M. Kimori Y. (2019). Autophagy controls reactive oxygen species homeostasis in guard cells that is essential for stomatal opening. *Proc. Natl. Acad. Sci. U.S.A.* 116 19187–19192. 10.1073/pnas.1910886116 31484757 PMC6754613

[B98] Yang C. Luo M. Zhuang X. Li F. Gao C. (2020a). Transcriptional and epigenetic regulation of autophagy in plants. *Trends Genet.* 36 676–688. 10.1016/j.tig.2020.06.013 32674948

[B99] Yang C. Shen W. Yang L. Sun Y. Li X. Lai M. (2020b). HY5-HDA9 module transcriptionally regulates plant autophagy in response to light-to-dark conversion and nitrogen starvation. *Mol. Plant* 13 515–531. 10.1016/j.molp.2020.02.011 32087368

[B100] Yoshimoto K. (2010). Plant autophagy puts the brakes on cell death by controlling salicylic acid signaling. *Autophagy* 6 192–193. 10.4161/auto.6.1.10843 20023431

[B101] Zhan N. Wang C. Chen L. Yang H. Feng J. Gong X. (2018). S-Nitrosylation targets GSNO reductase for selective autophagy during hypoxia responses in plants. *Mol. Cell* 71 142–154. 10.1016/j.molcel.2018.05.024 30008318

[B102] Zhang H. Si X. Ji X. Fan R. Liu J. Chen K. (2018). Genome editing of upstream open reading frames enables translational control in plants. *Nat. Biotechnol.* 36 894–898. 10.1038/nbt.4202 30080209

[B103] Zhang W. Gao S. Zhou X. Xia J. Chellappan P. Zhou X. (2010). Multiple distinct small RNAs originate from the same microRNA precursors. *Genome Biol.* 11 R81. 10.1186/gb-2010-11-8-r81 20696037 PMC2945783

[B104] Zhang Z. Zhu J. Y. Roh J. Marchive C. Kim S. K. Meyer C. (2016). TOR signaling promotes accumulation of BZR1 to balance growth with carbon availability in *Arabidopsis*. *Curr. Biol.* 26 1854–1860. 10.1016/j.cub.2016.05.005 27345161 PMC5126233

[B105] Zhong S. Fei Z. Chen Y. R. Zheng Y. Huang M. Vrebalov J. (2013). Single-base resolution methylomes of tomato fruit development reveal epigenome modifications associated with ripening. *Nat. Biotechnol.* 31 154–159. 10.1038/nbt.2462 23354102

[B106] Zhong X. Hale C. J. Nguyen M. Ausin I. Groth M. Hetzel J. (2015). Domains rearranged methyltransferase3 controls DNA methylation and regulates RNA polymerase V transcript abundance in *Arabidopsis*. *Proc. Natl. Acad. Sci. U.S.A.* 112 911–916. 10.1073/pnas.1423603112 25561521 PMC4311829

[B107] Zhou J. Wang J. Cheng Y. Chi Y. J. Fan B. Yu J. Q. (2013). NBR1-mediated selective autophagy targets insoluble ubiquitinated protein aggregates in plant stress responses. *PLoS Genet.* 9:e1003196. 10.1371/journal.pgen.1003196 23341779 PMC3547818

[B108] Zhou J. Wang J. Yu J. Q. Chen Z. (2014a). Role and regulation of autophagy in heat stress responses of tomato plants. *Front. Plant Sci.* 5:174. 10.3389/fpls.2014.00174 24817875 PMC4012191

[B109] Zhou J. Zhang Y. Qi J. Chi Y. Fan B. Yu J. Q. (2014b). E3 ubiquitin ligase CHIP and NBR1-mediated selective autophagy protect additively against proteotoxicity in plant stress responses. *PLoS Genet.* 10:e1004116. 10.1371/journal.pgen.1004116 24497840 PMC3907298

[B110] Zhu T. Li L. Feng L. Mo H. Ren M. (2020). Target of rapamycin regulates genome methylation reprogramming to control plant growth in *Arabidopsis*. *Front. Genet.* 11:186. 10.3389/fgene.2020.00186 32194640 PMC7062917

[B111] Zhu T. Zou L. Li Y. Yao X. Xu F. Deng X. (2018). Mitochondrial alternative oxidase-dependent autophagy involved in ethylene-mediated drought tolerance in *Solanum lycopersicum*. *Plant Biotechnol. J.* 16 2063–2076. 10.1111/pbi.12939 29729068 PMC6230944

[B112] Zhuang X. Chung K. P. Luo M. Jiang L. (2018). Autophagosome biogenesis and the endoplasmic reticulum: a plant perspective. *Trends Plant Sci.* 23 677–692. 10.1016/j.tplants.2018.05.002 29929776

