# Supplementary material for: Molecular Mechanisms of Autophagy Regulation in Plants and Their Applications in Agriculture
Source: Front Plant Sci. 2021 Feb 16;11:618944. doi: 10.3389/fpls.2020.618944 (PMC7921839; doi:10.3389/fpls.2020.618944)
Supplement: Supplementary Figure 1 — DNA methylation in plants. [file Data_Sheet_1.PDF]

Figure S1

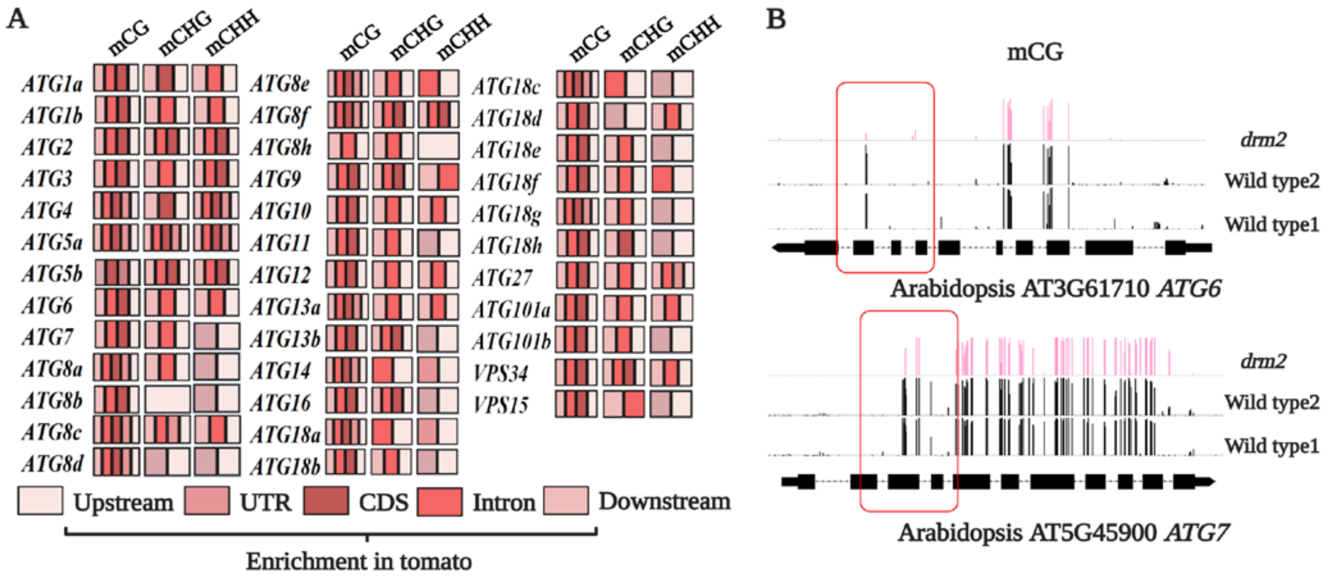
A
mCG
mCHG
mCHH
ATG1a
ATG1b
ATG2
ATG3
ATG4
ATG5a
ATG5b
ATG6
ATG7
ATG8a
ATG8b
ATG8c
ATG8d
ATG8e
ATG8f
ATG8h
ATG9
ATG10
ATG11
ATG12
ATG13a
ATG13b
ATG14
ATG16
ATG18a
ATG18b
ATG18c
ATG18d
ATG18e
ATG18f
ATG18g
ATG18h
ATG27
ATG101a
ATG101b
VPS34
VPS15
Upstream
UTR
CDS
Intron
Downstream
Enrichment in tomato
B
mCG
drm2
Wild type2
Wild type1
Arabidopsis AT3G61710 ATG6
drm2
Wild type2
Wild type1
Arabidopsis AT5G45900 ATG7

Supplementary Table 1. DNA methylation of Arabidospsis *ATG* genes

**Accession number**	**Gene name**	**mCG**	**mCHG**	**mCHH**
AT3G61960	*ATG1a*	Y	N	Y
AT3G53930	*ATG1b*	Y	Y	Y
AT2G37840	*ATG1c*	Y	N	N
AT1G49180	*ATG1t*	Y	N	Y
AT3G19190	*ATG2*	Y	Y	Y
AT5G61500	*ATG3*	Y	U	Y
AT2G44140	*ATG4a*	Y	N	U
AT3G59950	*ATG4b*	Y	N	Y
AT5G17290	*ATG5*	Y	U	Y
AT3G61710	*ATG6*	Y	N	Y
AT5G45900	*ATG7*	Y	U	Y
AT4G21980	*ATG8a*	N	N	U
AT4G04620	*ATG8b*	N	U	Y
AT1G62040	*ATG8c*	Y	N	Y
AT2G05630	*ATG8d*	Y	N	U
AT2G45170	*ATG8e*	N	N	N
AT4G16520	*ATG8f*	Y	N	U
AT3G60640	*ATG8g*	N	N	U
AT3G06420	*ATG8h*	N	N	U
AT3G15580	*ATG8i*	Y	N	U
AT2G31260	*ATG9*	Y	U	Y
AT3G07525	*ATG10*	N	N	U
AT4G30790	*ATG11*	Y	Y	Y
AT1G54210	*ATG12a*	N	N	U
AT3G13970	*ATG12b*	N	N	U
AT3G49590	*ATG13a*	Y	Y	Y
AT3G18770	*ATG13b*	Y	N	Y
AT1G77890	*ATG14a*	Y	N	U
AT4G08540	*ATG14b*	Y	N	U
AT5G50230	*ATG16*	Y	N	U
AT3G62770	*ATG18a*	Y	N	N
AT4G30510	*ATG18b*	Y	N	U
AT2G40810	*ATG18c*	Y	N	N
AT3G56440	*ATG18d*	Y	N	U
AT5G05150	*ATG18e*	N	N	Y
AT5G54730	*ATG18f*	Y	Y	Y
AT1G03380	*ATG18g*	Y	U	Y
AT1G54710	*ATG18h*	Y	Y	Y
AT2G40316	*ATG27*	Y	N	U
AT5G66930	*ATG101*	Y	N	U
AT4G29380	*VPS15*	Y	U	Y

AT1G60490	*VPS34*	Y	N	Y

Arabidopsis wild-type methylation data are derived from the whole-genome bisulfite libraries (Zhong *et al.*, 2015). Y, yes; N, no; U, uncertain.
